# Navigated minimally invasive puncture of the trigeminal cistern in horses—a cadaveric study in preparation for a controlled rhizotomy

**DOI:** 10.3389/fvets.2025.1562404

**Published:** 2025-06-13

**Authors:** Mathieu de Preux, Christina Precht, Richard Becker, Lennart Stieglitz, Jeremiah Easley, Christoph Koch

**Affiliations:** ^1^Division of Equine Surgery, Swiss Institute of Equine Medicine (ISME), Department of Clinical Veterinary Science, Vetsuisse-Faculty, University of Bern, Bern, Switzerland; ^2^Graduate School for Health Sciences, University of Bern, Bern, Switzerland; ^3^Division of Clinical Radiology, Department of Clinical Veterinary Science, Vetsuisse-Faculty, University of Bern, Bern, Switzerland; ^4^Department of Neurosurgery, University Hospital Zurich, Zurich, Switzerland; ^5^Preclinical Surgical Research Laboratory, Department of Clinical Sciences, Translational Medicine Institute, Colorado State University, Fort Collins, CO, United States

**Keywords:** trigeminal-mediated headshaking, rhizotomy, trigeminal cistern, trigeminal ganglion, neuronavigation, magnetic resonance tomography, cone beam computed tomography, postmortem angiography

## Abstract

**Introduction:**

Trigeminal-mediated headshaking is a neuropathic disorder in horses, characterized by signs of regional pain similar to trigeminal neuralgia in humans. The injection of glycerol into the trigeminal cistern to ablate pain-conducting nerve fibers within the trigeminal ganglion -known as glycerol rhizotomy- is a well-established treatment in human medicine. This study compares two approaches to the equine trigeminal cistern using a navigation system for guiding needle placement, a previously described ventral and a newly developed transmandibular lateral approach. The surgical accuracy and risk of iatrogenic collateral damage for the two approaches are assessed.

**Materials and methods:**

Five equine cadaveric specimens were used in this study. Magnetic resonance imaging (MRI) of the target region was performed using a 3 T MRI, followed by cone beam computed tomography (CBCT). The two datasets were fused in a surgical navigation system. A trajectory for a ventral navigated approach (VNA) to the trigeminal cistern was planned on one side and for a transmandibular lateral navigated approach (TLNA) on the contralateral side, using the system’s planning function. The trigeminal cistern was punctured after introducing the needle along the planned trajectory under real-time navigation guidance, and a toluidine blue solution was injected. A titanium rod was then inserted as a stylet to place a titanium seed within the trigeminal cistern. The rod was left in place to allow artifact-free postprocedural assessment of the surgical trajectory and to measure surgical accuracy aberration (SAA). Prior to dissection, an endoscopic examination was performed to identify any potential perforation of the guttural pouches.

**Results:**

Successful puncture of the trigeminal cistern was achieved in 5/5 specimens via the TLNA, with a median SAA of 3.92 mm (range 3.42 mm - 4.55 mm) and in 3/5 specimens via the VNA, with a median SAA of 6.45 mm (range 2.89 mm - 10.85 mm). The VNA resulted in iatrogenic injury to the internal carotid artery in two cases, and the linguofacial artery in another case. Focal perforation of the mucosa of the guttural pouch was observed in one specimen injected via TLNA.

**Conclusion:**

The TLNA enables accurate and precise minimally invasive puncture of the equine trigeminal cistern in an experimental setting.

## 1 Introduction

Trigeminal-mediated headshaking (TMHS) is a painful disorder of the horse characterized by violent, involuntary head and neck movements. Affected horses display signs of regional pain comparable to what is observed in people suffering from neuropathic facial pain caused by trigeminal neuralgia. Disease severity and clinical impact are highly variable, ranging from mild clinical signs under specific conditions to severe signs even at rest, possibly leading to early retirement or euthanasia in cases unresponsive to standard therapy (1).

Etiology and pathophysiology are currently not fully understood, but involvement of the trigeminal nerve has been implicated by assessment of sensory nerve conduction using somatosensory evoked potentials (2). The exact cause for aberrant trigeminal nerve activity remaining unknown, current management strategies and treatments are neither specific nor curative, and the condition carries a poor prognosis (1, 3). Different surgical interventions have been proposed to block the conduction of painful stimuli along the course of the trigeminal nerve in cases unresponsive to pharmacotherapy and symptomatic management (4–8). None of these treatments reliably achieves lasting pain relief, and the recurrence of clinical signs is common.

Trigeminal rhizotomy is a surgical technique that selectively destroys pain nerve fibers within the trigeminal ganglion. In humans with idiopathic trigeminal neuralgia and unresponsive to medical therapy, rhizotomy is performed using techniques such as balloon compression, glycerol rhizotomy, and radiofrequency thermocoagulation (9). The human trigeminal ganglion is embedded in a subarachnoid cistern within the trigeminal cave, also called the Meckel’s cave, which provides a well-defined anatomical compartment for targeted intervention (10). Rhizotomy is typically performed via percutaneous puncture of the trigeminal cave through the foramen ovale, under intermittent fluoroscopic guidance – also known as the Hartley technique (11). Recently, the microanatomy of the subarachnoid cistern of horses has been described (12). The equine trigeminal cistern represents the largest accumulation of cerebrospinal fluid in the subarachnoid space surrounding the trigeminal ganglion and plexus within the trigeminal cave. It is located dorsally and axially to the lateral edge of the foramen lacerum, remote from dense ganglionic tissue, and has a mean volume of approximately 0.3 mL. Accordingly, the equine trigeminal cistern could be targeted via a percutaneous approach through the foramen lacerum and injected with a predetermined amount of glycerol to perform rhizotomy.

Glycerol rhizotomy has been attempted in horses in an experimental setting and in clinical cases (13, 14). Using computed tomography (CT) guidance, this previously described ventral approach courses near the larynx, passing through the guttural pouch and the foramen lacerum. Due to inherently low soft-tissue resolution of CT imaging, potential iatrogenic injury to critical neurovascular structures cannot be reliably assessed and prevented, and accurate placement of the needle tip within the trigeminal cistern remains uncertain. In human medicine, percutaneous cannulation of the foramen ovale to access the trigeminal cave has similarly been associated with complications arising from inadvertent trauma to adjacent neurovascular structures (15, 16). Given the complication risk associated with the Hartley technique, which primarily relies on superficial anatomical landmarks and intermittent fluoroscopic guidance, and a reported technical failure rate of 1–5% (17), continuous efforts have been made to refine and improve the procedure (18). These refinements notably include the use of neuronavigation systems based on fused imaging datasets, which allow improved visualization of the trigeminal cistern and ganglion, enhance procedural navigation to minimize morbidity and avoid catastrophic complications, ultimately improving outcome of the rhizotomy procedure (17, 19, 20).

At the authors’ equine referral practice, a mobile cone beam CT (CBCT) unit is routinely used in conjunction with a navigation system employing optical tracking, providing real-time intraoperative guidance across a range of surgical procedures that require a high level of accuracy and precision (21–23). This technology has also been used for neuronavigated approaches by fusing CBCT with magnetic resonance imaging (MRI) datasets – for instance to access a pituitary macroadenoma in a mare via a transmandibular lateral approach (24). This novel transmandibular lateral navigated approach traverses the lateral pterygoid muscle in the roof of the guttural pouch and provides controlled access to the ventrolateral aspect of the equine cranium, while avoiding penetration of the guttural pouch and its potentially contaminated respiratory mucosa. This approach may therefore represent a safer alternative to the previously described ventral technique for minimally invasive puncture of the equine trigeminal cistern.

The primary objectives of the present study were to describe a minimally invasive, modified transmandibular lateral navigated approach (TLNA) to the trigeminal cistern and to compare it to a percutaneous ventral navigated approach (VNA) with regard to accuracy of needle placement and the potential for iatrogenic injury to vital structures.

We hypothesized that the TLNA would (A) have a shorter trajectory compared to the VNA, (B) enable more accurate and precise needle placement within the trigeminal cistern, (C) reduce the incidence of iatrogenic injury to the major branches of the common carotid artery, and (D) avoid perforation of the guttural pouch.

## 2 Materials and methods

**Figure 1:**
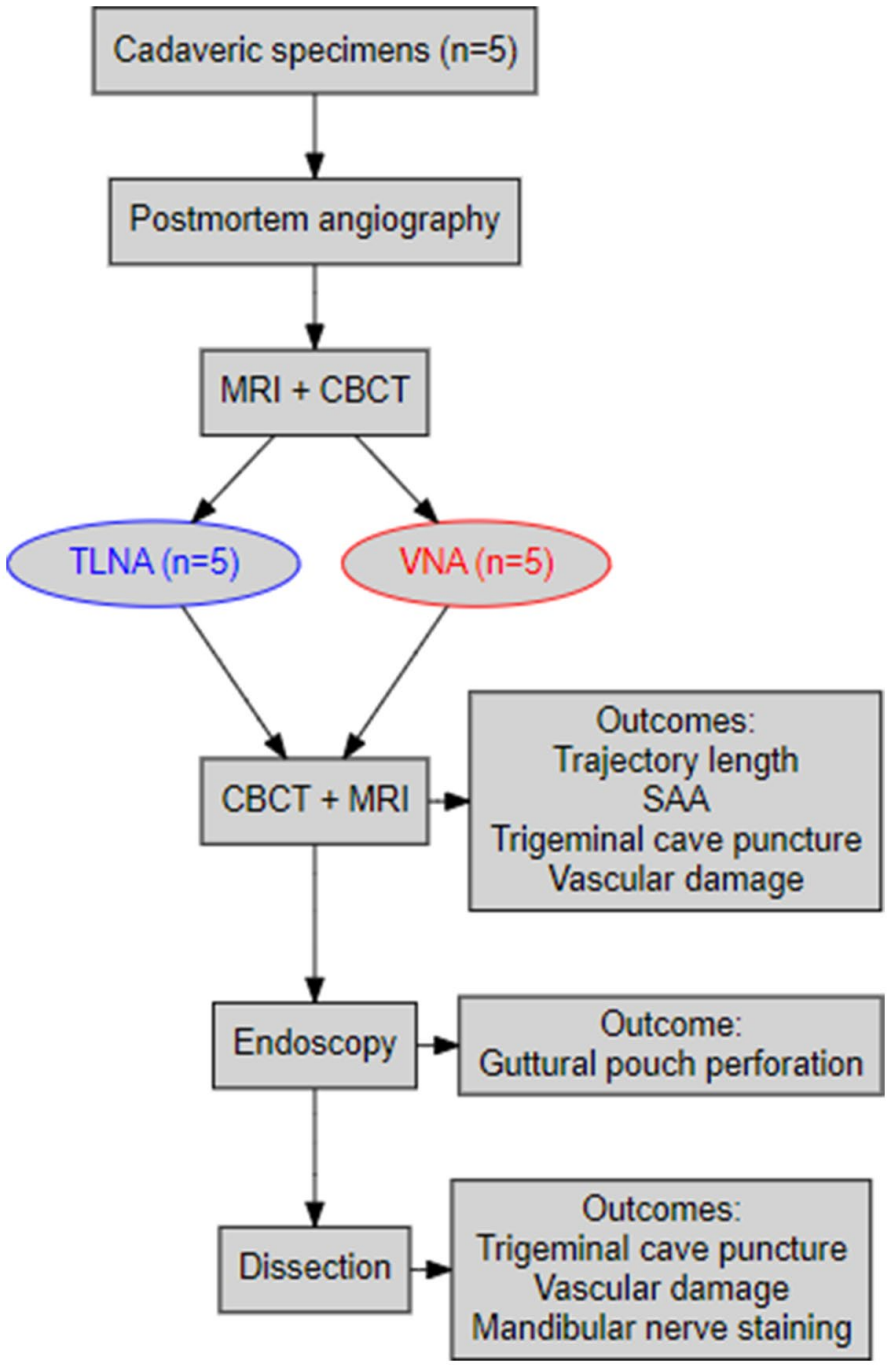
A flowchart summarizing the main procedural steps of the navigated minimally invasive punctures and the main outcomes of the study.

The steps of the experimental procedure are illustrated in a flowchart (Figure 1).

### 2.1 Cadaveric specimens

The horses providing the cadaveric specimens had no known history of TMHS or central nervous disease and were euthanized for reasons unrelated to the topic of this study. Euthanasia was performed via intravenous injection of pentobarbital (Esconarkon ad us. vet., Streuli Pharma AG, Uznach, Switzerland). Owners donated the cadaver for experimental research or teaching purposes by signing an informed consent form. Only specimens obtained from mature horses with a body weight of approximately 600 kg were included.

### 2.2 Preparation of cadaveric specimens and postmortem angiography

A postmortem angiography was performed to improve the visibility of the main branches of the common carotid arteries. Immediately following euthanasia, each cadaveric specimen was suspended by its legs on a hoist with the neck and head in the most dependent position to ensure blood filling the vascular system of the head. Both external jugular veins were dissected in the cranial third of the neck, and two circumferential ligatures with USP 2 polyglactin 910 (Vicryl Plus, Ethicon, Johnson & Johnson, Zug, Switzerland) were applied on each vein to avoid exsanguination of the venous system of the head. The neck was subsequently transected proximal to the ligated jugular veins, approximately at the level of the fourth cervical vertebra. To seal the vascular system, the musculature on the cut section of the neck and the vertebral canal were cauterized with a red-heated purpose-built iron plate. The left and right common carotid arteries were catheterized with a 4 mm inner diameter rigid plastic tube, mounted with a Luer-to-tubing connector. The catheters and attached tubing system were connected to a roller pump (DosiPump DP 1000 BioToolSwiss AG, Kirchberg, Switzerland) and the arterial system was perfused with highly viscous liquid paraffin (TRM-Ireland, Newbridge Industrial Estate, Co. Kildare, Ireland), at a rate of 100 mL/min for 2 min. Paraffin leakage observed from insufficiently cauterized vessels on the cut section of the neck was limited as much as possible by clamping those vessels with commercially available plastic clips (single use tube- and dialysis clamp, Fuhrmann GmbH, Much, Germany). Finally, the upper and lower incisors were firmly fixed together using a plastic cable tie passed through 4.5 mm drill holes prepared between the left and right second and third incisors to avoid alteration of the spatial relationship between the upper and lower jaws during the experimental procedure.

### 2.3 Preprocedural imaging

Immediately following cadaveric specimen preparation, as described above, each cadaveric specimen (i.e., the head and the upper third of the neck) was placed in a supine position within the gantry of a 3 Tesla MRI unit (Magnetom Vida, Siemens Healthcare AG, Zürich, Switzerland). Acquired sequences included a high-resolution dorsal 3D reconstructable magnetization-prepared gradient-echo (3D MP-RAGE) sequence with a voxel size of 0.5 × 0.5 × 0.7 mm^3^. All sequences included the brain and the surrounding soft tissue and bony structures of the head (i.e., the guttural pouches and the associated vasculature, the larynx, the masticatory muscles, and the vertical branches of the mandible). A board-certified radiologist (CP) reviewed the images for appropriate quality and potential artifacts caused by the liquid paraffin within the vascular system and to ensure that the area of interest (i.e., the trigeminal) was included and free of pathological findings. The cadaveric specimen was then moved out of the MR gantry and secured to a purpose-built wooden platform in supine position using commercially available tie-down straps. It was then placed on a carbon-fiber table (Opera Swing; General Medicale Merate, Seriate, Italy) and transferred into the gantry of a CBCT unit (O-arm 2, Medtronic, Louisville, Colorado, USA) that was coupled with a surgical navigation system with optical tracking (StealthStation S8; Medtronic). In each specimen, both trigeminal caves were punctured, first via a TLNA and subsequently via a VNA. The side of the head to be approached first was determined for each specimen by the toss of a coin.

A patient tracker (passive orthopedic reference frame 963–864 and fixator 9,730,864, StealthStation System, Medtronic) was anchored using two 3.2 mm self-tapping Schanz pins inserted through stab incisions on the facial crest of the respective side (left or right) on which the procedure was performed. The cadaveric specimen stabilized on the wooden platform was brought into the center of the CBCT gantry, and two orthogonal fluoroscopic images were acquired to ensure that the targeted foramen lacerum was positioned in the center of the field of view. A standard 3D CBCT scan (i.e., 192 images during one tube rotation with an exposure of 120 kV and 20 mA, and a voxel size of 0.4 × 0.4 × 0.8 mm^3^) was then acquired and automatically transferred to the surgical navigation system. During the CBCT scan, the localizer camera of the optical tracking system simultaneously detected the reflecting spheres of the patient tracker and the infrared light-emitting tracker of the CBCT gantry. The previously acquired dorsal 3D MP-RAGE MRI sequence was manually imported from the picture archiving and communication system (PACS, DeepUnity Diagnost, Version 2.0.2.2, Dedalus Healthcare Group, 2024) into the navigation system (StealthStation SS8, Medtronic) and fused with the CBCT-scan using the StealthMerge function of the Cranial Software (both Medtronic), providing a complete image volume for neuronavigation.

### 2.4 Minimally invasive punctures

#### 2.4.1 Transmandibular lateral navigated approach (TLNA)

**Figure 2:**
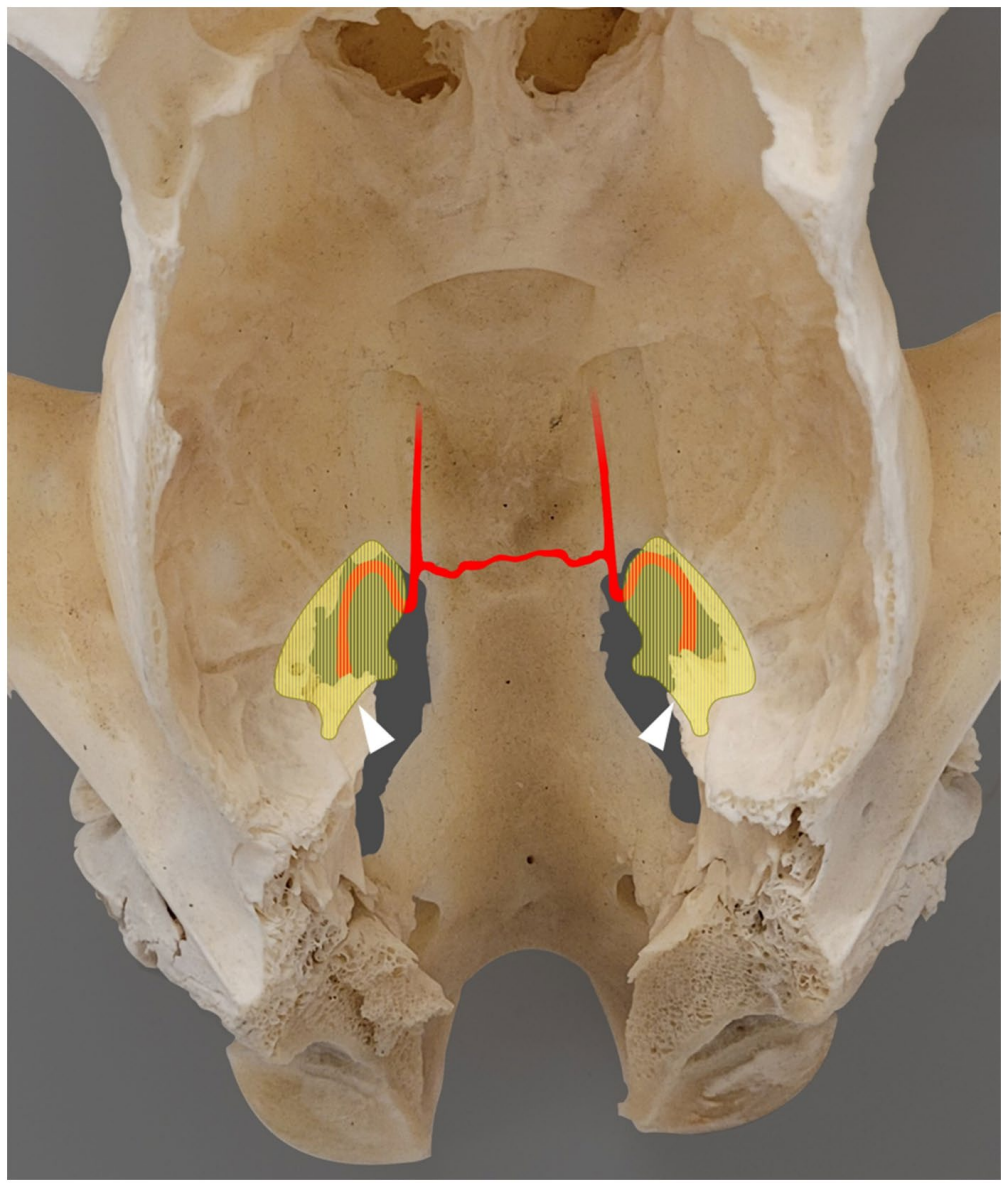
Photograph showing a dorsal view of the skull of a skeletally mature horse, following removal of the calvaria and all soft tissue structures. The areas shaded in light yellow indicate the left and right trigeminal cisterns, partially superimposed with the corresponding foramen lacerum [based on microtomographic data from Becker et al. (12)]. Rostral is on the top of the image. The internal carotid artery enters the foramen lacerum at the lateral carotid incisure, ventral to the trigeminal cistern, and then forms a sigmoid flexure at the rostral margin of the foramen lacerum. The two internal carotid arteries are interconnected via the intercarotid artery. The white arrowhead indicates the approximate entry point of the trigeminal nerve into the trigeminal cistern through the porus trigeminus.

**Figure 3:**
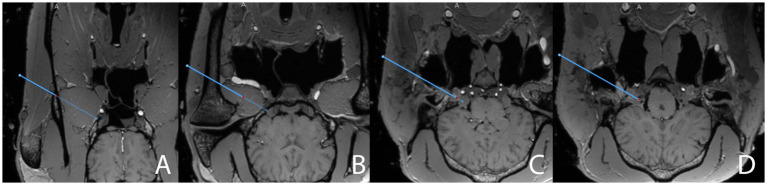
Close-ups of screenshots from the surgical navigation system (StealthStation S8) sequentially depicting the surgical planning (blue line) in the transverse plane for a transmandibular lateral navigated approach (TLNA) to the trigeminal cistern on merged T1-weighted sequence (3D MP-RAGE)- and CBCT datasets. The full surgical planning across all three anatomical planes is shown in “Supplementary Video S1.” Please note the hyperintense signal caused by the paraffin oil filling the main branches of the common carotid artery in the cadaveric specimen. The position of the red dot on the blue line representing the surgical plan is assessed to ensure that the surgical plan does not interfere with critical anatomical structures. The plan **(A)** crosses the vertical ramus of the mandible, just dorsal to the maxillary vein, **(B)** then runs through the lateral pterygoid muscle, at a reasonable distance from the maxillary artery and the roof of the guttural pouch, **(C)** enters the cranial cavity through the foramen lacerum while “flying over” the sigmoid flexure of the internal carotid artery (hyperintensity located above the red dot), and **(D)** finally reaches the trigeminal cistern (hypointense spot on the axial aspect of the trigeminal ganglion in the trigeminal cave, located above and to the right of the red dot in the image).

Planning and puncture were always performed by the same investigator (MdP). Planning was performed on the fused MR- and CBCT-dataset using the planning function of the Cranial Software (Medtronic). First, the target point was set within the trigeminal plexus, i.e., the caudal half of the trigeminal cave, slightly caudal and axial to the trigeminal ganglion, where the largest accumulation of cerebrospinal fluid was visible on the 3D MP-RAGE sequence. The trajectory for the TLNA was then defined using the orientation provided by the multiplanar reconstructions of transverse, dorsal, and sagittal planes on the fused images. Patient registration was performed by contacting the divot of the patient tracker with a navigated pointer (Passive Planar Probe [sharp], 960–553, StealthStation System, Medtronic). The navigated pointer was then used to define the appropriate trajectory by contacting the surface of the skin of the cheek with its tip, ventral to the temporomandibular joint, and by aiming towards the predefined target point in the trigeminal cave with its virtual projection displayed on the monitor of the navigation system. Invariably, the trajectory passed through the vertical ramus of the mandible and the lateral pterygoid muscle to gain access to the foramen lacerum without perforating the guttural pouch. Planning the trajectory, special care was taken to avoid the large maxillary vein on the axial aspect of the vertical ramus of the mandible, the maxillary artery in the roof of the lateral compartment of the guttural pouch, the axial aspect of the mandibular condylar process, the sigmoid flexure of the internal carotid artery (ICA) at the level of the foramen lacerum, and the edges of the foramen (Figures 2, 3, Supplementary Video S1). Following initial planning, the probe’s eye function of the navigation system was repeatedly run to critically assess the course of the planned trajectory. By slightly adjusting the position of the entry point, the course of the trajectory was revised as needed, until it was in a safe corridor to the trigeminal cave.

The navigated pointer was then used to identify the planned entry point for the puncture. Centered on this point, the skin and superficial fascia of the underlying masseter muscle were sharply incised over a length of approximately 3 cm, in an orientation that would allow preserving the integrity of the main branches of the facial nerve. The muscle fibers of the exposed masseter were bluntly separated to gain access to the surface of the vertical ramus of the mandible. A lockable stereotactic aiming device (Vertek aiming device (VAD), Medtronic) for rigid instrument guidance (25) was then anchored to the corresponding edge of the wooden platform. The navigated pointer was inserted through a custom-made 4.0 mm reducing sleeve into the drill-guide barrel of the VAD. The long axis of the drill-guide barrel was then aligned with the surgical plan by using the projection of the navigated pointer and guidance provided by the navigation system. The VAD was locked in place once a target alignment error smaller than 1 mm was achieved. The trajectory length (in mm) was protocolled, and the navigated pointer and its corresponding reducing sleeve were replaced by a 3.2 mm high-speed drill bit (Midas Rex Legend, DS1TD32L, Medtronic). The drill handpiece fitted snugly in the drill-guide barrel of the VAD. Therefore, the long axis of the drill bit was aligned with the surgical plan and was used to accurately perform a minimally invasive circular osteotomy in the vertical ramus of the mandible along the planned trajectory.

**Figure 4:**
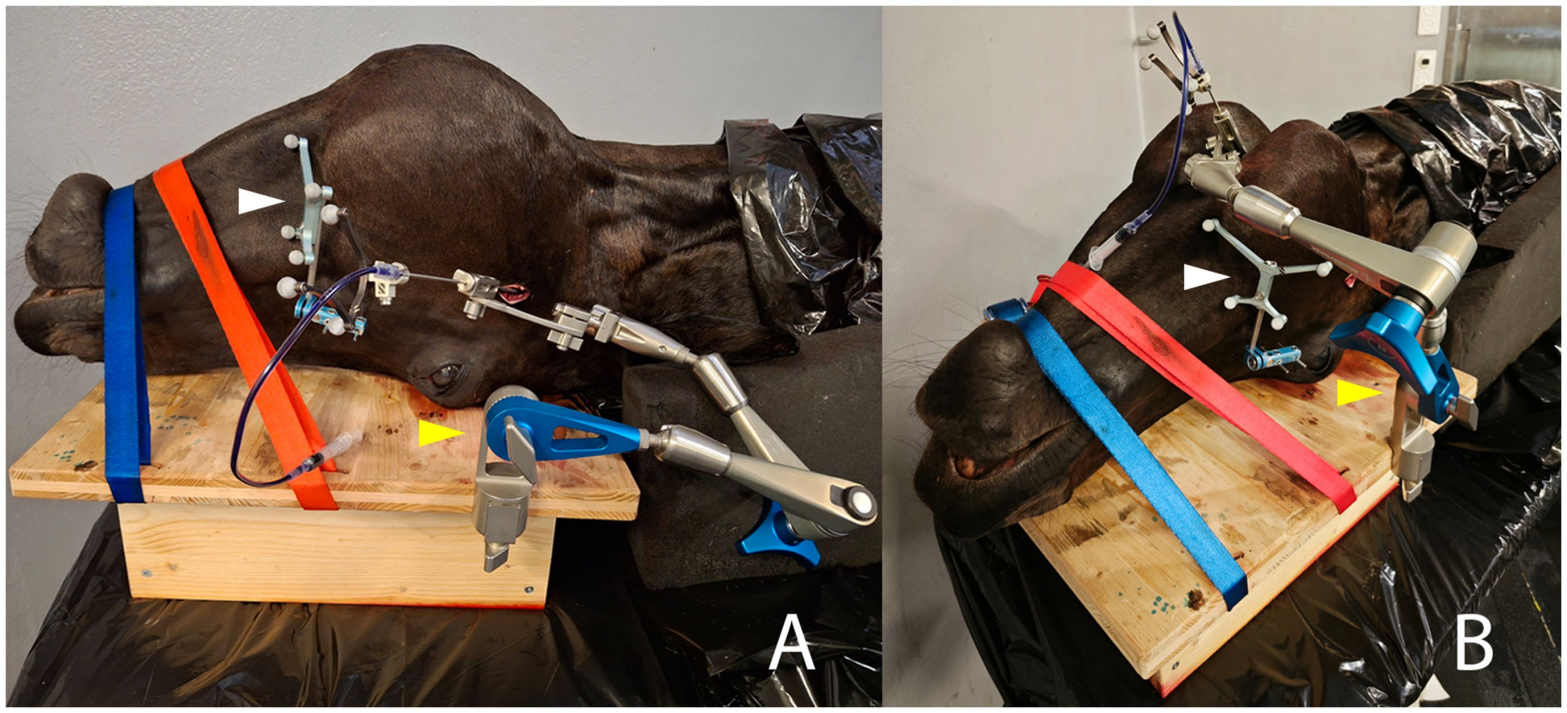
Intraprocedural photographs illustrating the two compared approaches for minimally invasive puncture of the right trigeminal cistern in the same equine cadaveric specimen. The patient reference tracker (white arrowhead) is anchored on the right facial crest using two 3.2 mm Schanz pins. The cadaveric specimen is stabilized with two straps on a wooden board, to which the Vertek aiming device (yellow arrowhead) is attached. **(A)** Lateral transmandibular navigated approach: the navigated needle, mounted with a black instrument tracker, is inserted through the corresponding sleeve in the aiming device, and an incision is made in the right cheek/masseter muscle. **(B)** Ventral navigated approach: the navigated needle is again inserted through the corresponding sleeve of the aiming device and through a skin incision located about 2 cm rostral to a line running through the caudal aspect of the vertical ramus of the mandible and 1 cm axial to the horizontal ramus of the mandible.

An 18 G, 335 mm long aspirating needle (DTR Medical, Swansea, UK), mounted with an instrument tracker (SureTrak II Universal Tracker, Large Passive Fighter, 961–581, Medtronic), was calibrated and prepared for the minimally invasive puncture. A 50 cm long and 0.88 mm diameter rod of grade 5 titanium was introduced as a stylet through the sharp end of the needle and pushed towards its hub to leave sufficient space to accommodate for a 2 mm long, 0.88 mm diameter titanium seed which was subsequently loaded within the tip of the needle. A 30 cm perfusion extension line, connected to a 2 mL syringe, was threaded over the titanium stylet protruding from the hub, attached to the Luer hub of the needle, and filled with a solution of water and toluidine blue. The navigated and loaded needle was subsequently introduced in a custom-made 3.5 mm reducing sleeve (corresponding to its external diameter) placed in the barrel of the VAD (Figure 4A). The needle was then carefully advanced through the osteotomy site, the lateral pterygoid muscle, and the cartilage covering the foramen lacerum while constantly monitoring its penetration depth and alignment with the planned trajectory on the screen of the navigation system. If the needle had to be fully or partially withdrawn to adjust its orientation, the number of attempts were documented. Once the needle tip reached the target point, a CBCT scan was run with the needle in place, and the images were merged with the preprocedural CBCT scan to allow for the measurement of surgical accuracy aberration (SAA). Subsequently, 0.5 mL of toluidine blue mixture was injected, and the needle was carefully withdrawn while an assistant maintained the titanium rod in position. This deployed the titanium seed and the titanium stylet, marking the trajectory of the needle for a later artifact-free assessment. A final postprocedural CBCT scan was then run and merged with the previously gathered datasets before the titanium stylet was removed, leaving only the implanted titanium seed behind.

#### 2.4.2 Ventral navigated approach (VNA)

**Figure 5:**
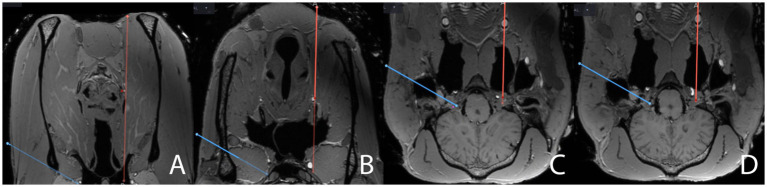
Close-ups of screenshots from the surgical navigation system sequentially depicting the surgical planning (red line) in the transverse plane for a ventral navigated approach (VNA) to the trigeminal cistern on the same merged MRI- and CBCT-datasets, augmented by postmortem angiography, as in Figure 2. The full surgical planning across all three anatomical planes is shown in “Supplementary Video S2.” The plan for a transmandibular lateral navigated approach (TLNA, blue line) is still shown on the images for comparison purposes. The position of the red dot on the red line representing the surgical plan is assessed to ensure that the surgical plan does not interfere with critical anatomical structures. The plan for a VNA **(A)** starts just axially to the ventral aspect of the mandible, close to the linguofacial vein, **(B)** passes close to the larynx and the linguofacial artery, **(C)** perforates the guttural pouch and its roof, where it crosses the sigmoid flexure of the internal carotid artery, and **(D)** enters the cranial cavity through the foramen lacerum to finally reach the trigeminal cave. Please note that, when planning the VNA, the target point in the trigeminal cave cannot be set as axially as when planning the TLNA, as this would result in interference of the plan with the internal carotid artery.

Following completion of the TLNA, the patient tracker was anchored on the contralateral facial crest and a CBCT scan was acquired. Planning for the VNA was performed, as described above for the TLNA. Again, the target was set within the trigeminal plexus as described for the contralateral side. However, the planned entry point of the trajectory was now set about 2 cm rostral to a line running through the caudal aspect of the vertical ramus of the mandible and 1 cm axial to the horizontal ramus of the mandible, as described elsewhere (13). This invariably resulted in a trajectory coursing through the medial compartment of the guttural pouch. When planning the ventral trajectory, special care was taken to avoid the linguofacial artery (a branch of the external carotid artery situated in close vicinity to the larynx), the laryngeal cartilages, the hyoid apparatus, the sigmoid flexure of the ICA in the roof of the guttural pouch, and the edges of the foramen lacerum (Figure 5, Supplementary Video S2). To carefully assess the trajectory of the plan, the probe’s eye function was used, and adjustments were made as necessary before finalizing the plan.

A longitudinal, approximately 3 cm skin incision was centered over the planned entry point (identified with the navigated pointer), avoiding the linguofacial vein. Again, the VAD was anchored on the corresponding edge of the wooden platform, and the drill-guide barrel was aligned with the surgical plan using the navigated pointer. Once satisfactory alignment was achieved, the VAD was locked in place, and the plan was finalized if a target alignment error smaller than 1 mm was reached. The length of the trajectory was protocolled. The aspirating needle was prepared as described for the TLNA and inserted under navigation guidance until its tip reached the target point (Figure 4B). The number of attempts was documented. The same postprocedural imaging protocol as for the TLNA was repeated (i.e., CBCT scan with the needle in place, second CBCT scan only with titanium rod and seed, merging of all the scans) before removing the titanium rod.

### 2.5 Assessment of the surgical accuracy and success of the puncture

**Figure 6:**
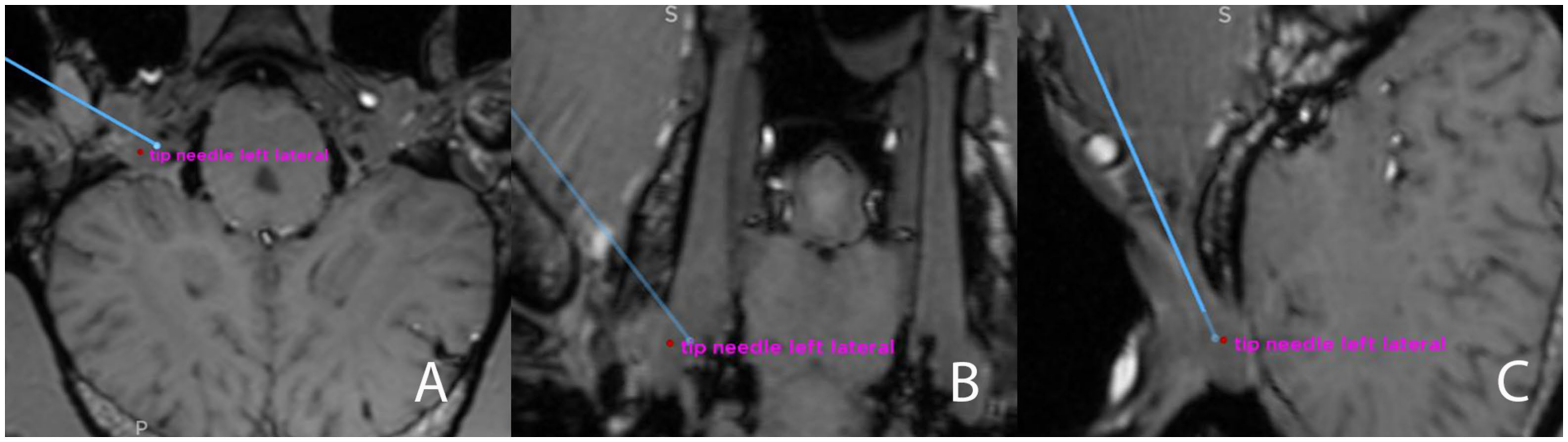
Screenshot from the surgical navigation system showing pre- and postprocedural merged T1-weighted sequence (3D MP-RAGE)- and CBCT datasets. The blue line represents the surgical plan for a transmandibular lateral navigated approach to the trigeminal cistern, in all three anatomical planes **(A)** transverse; **(B)** dorsal; **(C)** sagittal. The endpoint of the blue line is the planned target, and an annotation (red dot) has been set at the tip of the needle (i.e., the achieved target, “tip needle left lateral”) The Euclidean distance between those two points will be calculated after exporting the coordinates.

After completing both punctures, annotations were manually set at the tip of both inserted needles on the fused pre- and post-procedural CBCT dataset (Figure 6). Patient coordinates, including each surgical plan’s (planned) target point, and the annotations (i.e., the achieved targets) were subsequently exported from the navigation system. For each procedure, the surgical accuracy aberration (SAA, in mm), defined as the Euclidean distance between the intended and achieved target, was then calculated using the formula: error = √[(x2-x1)^2^ + (y2-y1)^2^ + (z2-z1)^2^], where x1, y1, and z1 represents the coordinates of the planned target, and x2, y2 and z2 the coordinates of the achieved target.

**Figure 7:**
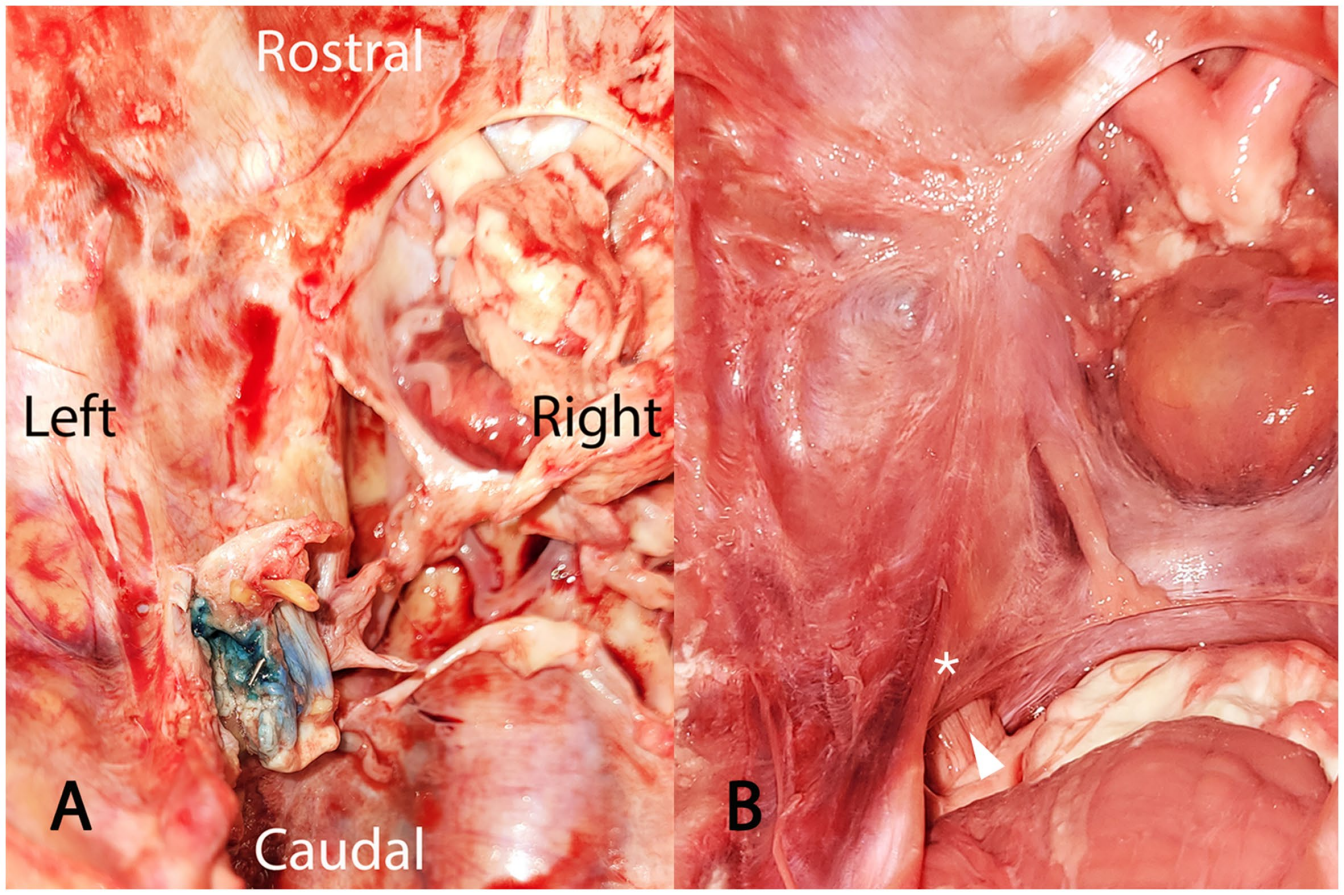
Photograph showing **(A)** a dissected left trigeminal cave, viewed from dorsal, after removal of the calvaria, the cerebrum, the pons, and the cerebellum. On this cadaveric specimen, puncture of the trigeminal cistern was successful, as indicated by the toluidine blue staining of the trigeminal plexus (recognizable by its typical trabecular appearance) and the presence of the titanium seed within this structure. For comparison, a photograph **(B)** of a normal, non-dissected left trigeminal cave, also viewed from dorsal. The cerebrum has been removed. The optic chiasm and the pituitary gland are visible at the top of the image. The cerebellum and the pons are visible at the bottom of the image. The trigeminal nerve (white arrowhead), originating from the pons, enters the trigeminal cave through the porus trigeminus (please refer to Figure 2 for a schematic visualization of the spatial relationship between the trigeminal cistern and the bony edges of the foramen lacerum). The white asterisk is positioned above the dura mater lining the trigeminal cave, which would correspond to the approximate location of the titanium seed shown in **(A)**.

Successful puncture of the trigeminal cistern was defined as the detection of a fluid artifact within the trigeminal plexus and a slight artifact caused by the titanium seed on the postprocedural MRI sequences, and the detection of blue staining of the trigeminal plexus and location of the titanium seed within the trigeminal plexus on dissection (Figure 7).

### 2.6 Assessment of iatrogenic damages

#### 2.6.1 Magnetic resonance imaging

The cadaveric specimen was again placed in a supine position within the gantry of the MRI, and the same sequences as before the procedure were acquired. The exact location of the slight artifact induced by the implanted titanium seed and by the injected toluidine blue solution was determined on the MR images. The postprocedural dorsal 3D MP-RAGE sequence was manually imported into the navigation system and fused with the (already fused) preprocedural MP-RAGE sequence and postprocedural CBCT dataset, to detect potential overlaps of the titanium rods with the main vessels (especially the maxillary vein, the linguofacial artery, the maxillary artery, and the ICA), thus allowing for assessment of iatrogenic damage to the vasculature.

#### 2.6.2 Endoscopy

Subsequently, a transnasal endoscopy of the left and right guttural pouches was performed with the cadaveric specimen in a prone position, using a 1.40 m long, 7.9 mm diameter, flexible endoscope (Storz AG, Tuttlingen, Germany), to identify potential perforations of the guttural pouch and iatrogenic damage to associated neurovascular structures.

#### 2.6.3 Anatomical dissection

Each cadaveric specimen was dissected directly following the experimental procedure. The head was disarticulated at the atlantooccipital joint, and the lower jaw was removed to access the roof of the guttural pouch and the ventral aspect of the foramen lacerum. The mandibular nerve of each side was identified and examined for toluidine blue staining. Following the removal of the calvaria with an oscillating saw, the cerebrum was removed using a combination of blunt and sharp dissection to expose the cerebellum, the pons, and the trigeminal roots. The trigeminal roots were sharply separated from the pons, and the cerebellum was removed. On each side, the trigeminal porus was identified, and the dura mater surrounding the trigeminal cave was carefully dissected to access the trigeminal plexus and ganglion, the ICA perforating the cartilage covering the foramen lacerum at the lateral carotid incisure, and the intercarotid artery in the epidural space. The integrity of the vascular structures was assessed. Furthermore, the presence and distribution of toluidine blue staining and the exact location of the titanium seed were documented.

### 2.7 Statistical analysis

The collected data were analyzed using R statistical software (v4.1.2; R Core Team 2021). The surgical accuracy aberrations of the TLNA and the VNA were compared using descriptive statistics, and a scatter plot was obtained using the ggplot2 R package (v3.3.3) (26) and tidyr R package (27).

## 3 Results

### 3.1 Study population

Five cadaveric specimens were included, resulting in a total of ten punctures targeting the trigeminal cistern, i.e., five via a TLNA and five via a VNA. Two VNA were performed on the left and three on the right side. The cadaveric specimens were harvested from 3 Warmblood horses, one Andalusian horse, and one Black Forest horse. The age of the horses ranged from 5 to 19 years (median 15), and the body weight ranged from 570 to 674 kg (median 611 kg).

### 3.2 Image quality

The liquid paraffin was visible as a hyperintense fatty signal on the 3D MP-RAGE sequence within the lumen of the common carotid arteries, the internal and external carotid arteries, and their branches including the maxillary and the linguofacial arteries. As expected, no distribution of paraffin was visible within the venous system due to the inability of this highly viscous liquid to pass through the capillary system. No paraffin extravasation was noticed, and no artifact precluded interpretation of the MR images. In one cadaveric specimen, filling defects caused by intravascular persistence of blood clots were visible in the internal and external carotid artery. The paraffin perfusion on this cadaveric specimen was performed with a delay of a few hours after euthanasia. Although not ideal, this did not preclude appropriate identification of the relevant vasculature.

### 3.3 Trajectories

The median length of the VNA was 220 mm (range 164 mm – 233 mm), and the median length of the TLNA was 137 mm (range 108 mm – 175 mm). In every specimen, the VNA was longer than the TLNA.

In all TLNAs, the osteotomy site of the vertical ramus of the mandible was located approximately 3 cm ventral to the mandibular notch (which is the groove between the mandibular condylar process and the coronoid process) and midway between the rostral and caudal edge of the vertical ramus.

In all VNAs, the needle crossed the stylohyoid bone on its axial side and, therefore, passed through the medial compartment of the guttural pouch. The perforation site of the needle through the roof of the guttural pouch was identified on endoscopy as a focal mucosal lesion located just rostral to the temporohyoid joint and was consistently associated with a variable amount of blood contamination of the medial compartment of the guttural pouch.

### 3.4 Number of attempts

In 8/10 punctures (4/5 for VNA and 4/5 for TLNA), only one attempt was necessary to insert the needle along the planned trajectory and reach the planned target. Using the VNA, the needle had to be redirected in one specimen due to interference with the larynx, which precluded further advancement. Furthermore, the needle tip interfered with the edge of the foramen lacerum in another specimen when taking the VNA. Despite this, the needle could still be advanced, but the titanium seed could not be deployed because the needle tip was bent, so the titanium stylet could no longer be advanced. Using the TLNA, redirection of the needle was necessary in one specimen due to interference of the tip of the needle with the axial aspect of the mandibular condylar process.

### 3.5 Iatrogenic damages to vascular structures

As previously described, the VNA invariably ran near the larynx and the associated vasculature. Interference of the titanium stylet with the large linguofacial artery was identified on the postprocedural fused images in one specimen and was strongly suspected in another. Although special care was taken to avoid the ICA and its sigmoid flexure when planning the trajectory, the VNA resulted in iatrogenic injury to this structure in two cases, as detected on the fused postprocedural images and during the dissection. In two additional specimens with a VNA, the needle was inserted close (within < 1 mm distance) to the ICA. The TLNA did not result in iatrogenic damage to either the ICA or the maxillary artery. However, in one specimen with a TLNA, the needle was inserted close to the maxillary artery in the roof of the guttural pouch and lacerated the maxillary vein. In this specimen, the endoscopy of the guttural pouch revealed two focal mucosal lesions just lateral to the temporohyoid joint, at about 1 cm distance to each other, which were highly suggestive of iatrogenic perforation. In all other specimens, the needle paths of the TLNAs were entirely located within the lateral pterygoid muscle and, therefore, did not perforate the guttural pouch.

### 3.6 Surgical accuracy and success of the puncture

**Figure 8:**
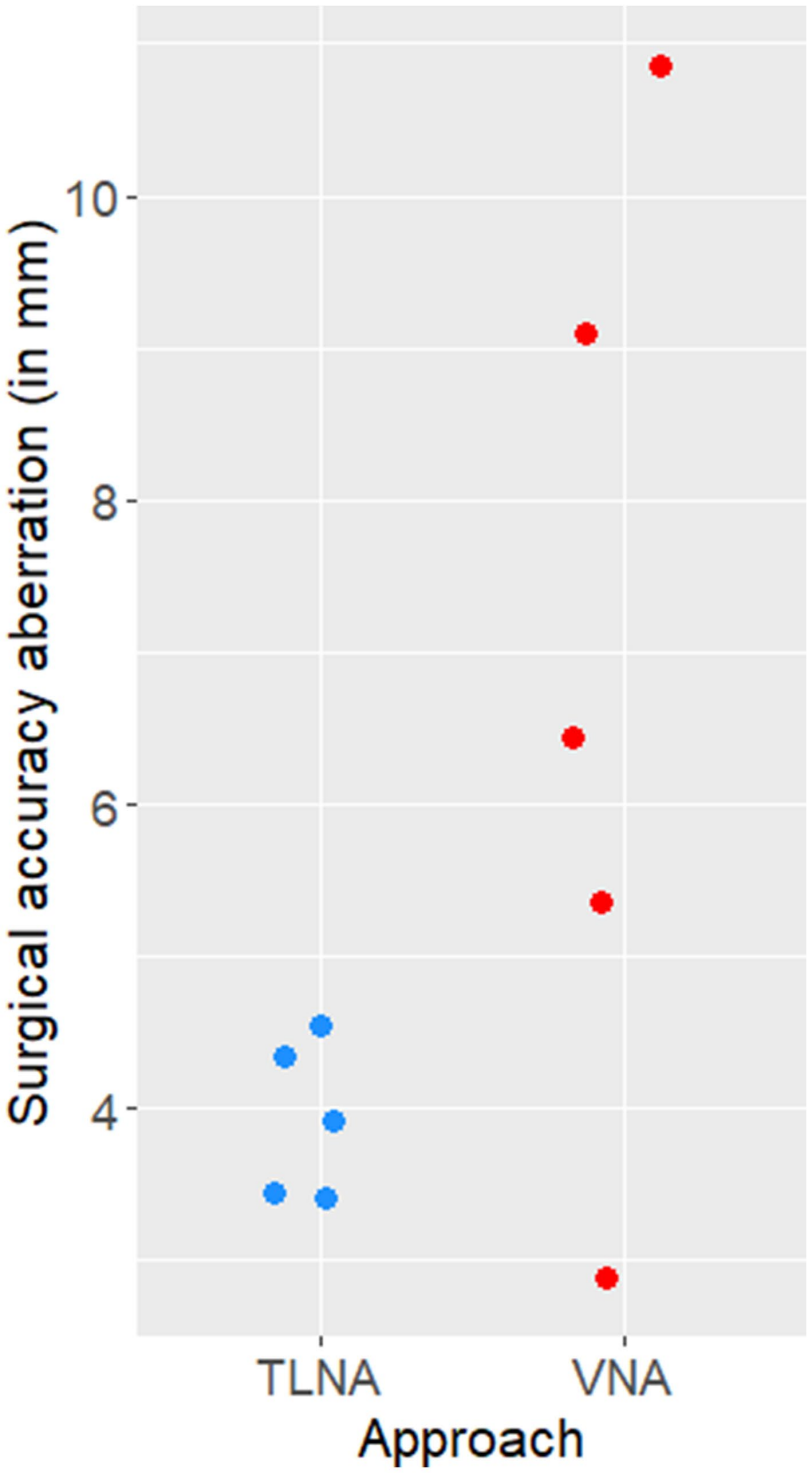
Scatter plot illustrating the surgical accuracy aberration (Euclidean distances, in mm) of needle placement achieved on the five specimens when using the transmandibular lateral navigated approach (TLNA) versus the ventral navigated approach (VNA). Please note that the Euclidean distances resulting from the TLNA are clustered around a low value (4 mm) and have a narrow distribution (i.e., high surgical accuracy and precision).

The median SAA (Euclidean distances) of the achieved target was 6.45 mm (range 2.89 mm - 10.85 mm) when performing the VNA and 3.92 mm (range 3.42 mm - 4.55 mm) when performing the TLNA (Figure 8).

Successful puncture of the trigeminal cistern was achieved in 5/5 TLNA and 3/5 VNA (total of 8/10 successful punctures). This includes one specimen with a VNA, in which the titanium seed could not be deployed successfully due to bending of the tip of the needle (see above). However, a fluid artifact was visible in this specimen on MRI, and the trigeminal plexus was stained blue, confirming the successful injection. In one unsuccessful VNA, the seed was inserted in the epidural space, indicating that the needle had perforated the cartilage covering the foramen lacerum but not the dura mater surrounding the trigeminal cave, which explains why the trigeminal plexus was not stained blue. In the other unsuccessful VNA, the needle interfered with the caudolateral edge of the foramen lacerum, failing to perforate its cartilage. This resulted in the deployment of the titanium seed just caudal and lateral to the foramen lacerum. Interestingly, in two cadaveric specimens where both trigeminal cisterns were successfully punctured, toluidine blue staining was observed along both mandibular nerves over a variable length.

## 4 Discussion

This experimental cadaveric study describes a novel approach for minimally invasive puncture of the trigeminal cistern in horses. Furthermore, the TLNA approach is compared to a previously described ventral approach to the trigeminal cistern (13). Regardless of the approach, the procedures were performed under navigation guidance. The TLNA resulted in higher surgical accuracy and precision in needle placement within the trigeminal cistern compared to the VNA. This consistently resulted in successful puncture of the targeted trigeminal cistern in all specimens, whilst avoiding iatrogenic injury to the major branches of the common carotid artery. However, a focal perforation of the roof of the guttural pouch was observed in one specimen, highlighting the importance of careful surgical planning to ensure that the entire trajectory remains within the lateral pterygoid muscle. Despite the use of advanced 3D imaging-based navigation, puncture of the trigeminal cistern was not consistently achieved using the VNA and was frequently associated with iatrogenic injury to the sigmoid flexure of the ICA.

In the present study, two key factors were identified as critical for achieving accurate and precise puncture of the trigeminal cistern within the trigeminal cave, while avoiding major surrounding vascular structures: (A) the use of a surgical navigation system with optical tracking, enabling real-time monitoring of needle orientation and penetration depth, and (B) the fusion of CBCT images with preprocedural MRI and angiography, providing detailed spatial information of critical neurovascular structures.

In human medicine, the use of real-time fusion imaging guidance for percutaneous cannulation of the foramen ovale has been shown to improve both cannulation success rate and needle placement accuracy within the trigeminal cistern (19). A previous report on glycerol rhizotomy in horses (13) described the use of sequential intraoperative CT imaging for guidance and confirmation of needle placement within the trigeminal cave, but without the aid of a computer-assisted navigation system. The results of the present study highlight the importance of accurate placement of the needle tip within the trigeminal cave to ensure effective delivery of liquid agents such as glycerol to the trigeminal ganglion. The needle tip was inserted into the largest accumulation of cerebrospinal fluid in the trigeminal cave, which is invariably axial and caudal to the dense trigeminal ganglionic tissue and well visible on the MRI sequence used for navigation. Given the small diameter of the nerve bundles forming the loose trigeminal plexus, the larger size and beveled shape of the needle tip, and because of the postprocedural uniform, broad blue staining of the trigeminal plexus, it seems likely that the tip was located within the trigeminal cistern. Postprocedural histological examination of the trigeminal cave could confirm injection into the subarachnoid space and not into the neural tissue itself. However, this still would not predict the efficacy of the procedure in live animals, which is why we decided not to investigate this point further in this cadaveric study.

Given the presumably narrow margin of error for performing a rhizotomy of the trigeminal ganglion safely and effectively in horses, the use of neuronavigation (preferentially in combination with the novel TLNA) appears to be the minimal prerequisite to attempt this procedure in a clinical setting. In seven out of eight specimens, the trigeminal cave was successfully approached with a single attempt, similar to what is reported for cannulation of the foramen ovale under navigation guidance in humans (20). This markedly contrasts the previous report on glycerol rhizotomy in horses, where a mean of four attempts was necessary to reach the trigeminal ganglion, highlighting the hazardous nature of the procedure. Indeed, with each attempt and repositioning of the needle, the risk of complications arising from iatrogenic trauma to vital neurovascular structures increases. Furthermore, repeated intraoperative CT imaging exposes neurosensory tissue to higher cumulative radiation compared to a single preprocedural CBCT required for neuronavigated percutaneous puncture (17).

While the proposed neuronavigation set-up seems promising for minimally invasive puncture of the trigeminal cistern via the TLNA, the same cannot be stated for the VNA. Based on the findings of the present study, the VNA has a lower rate of successful puncture, higher overall SAA, and higher variability in SAA compared to the TLNA. These results, together with the high incidence of iatrogenic injury to vascular structures with the VNA, clearly support the TLNA as the preferred approach. Several factors may account for the greater surgical precision associated with the TLNA. The entry to target distance is shorter with the TLNA than the VNA, with a median of approximately 140 mm vs. 220 mm. It is reasonable to speculate that this difference significantly influences both accuracy and precision, especially when inserting a 30 cm long, semi-rigid needle. Bending of a navigated needle during its advancement toward the target invariably results in deviation from the planned trajectory (17). In the present study, the VAD was used to improve stability, reduce needle bending, and ensure more rigid (and thus accurate) guidance and alignment with the planned trajectory. The stabilizing effect of the aiming device is potentiated in the TLNA, where the osteotomy in the vertical ramus of the mandible provides a second fixed point for needle stabilization. Beyond the osteotomy, the needle is advanced through the belly of the lateral pterygoid muscle until it reaches the cartilage of the foramen lacerum. The muscle presents minimal resistance to needle passage, allowing controlled advancement. In contrast, the VNA requires the needle to traverse multiple tissue layers of different density and resistance, in close proximity to dense anatomical structures, such as the larynx and the hyoid apparatus, both of which can easily induce needle bending.

The SAA values reported in this study represent Euclidean distances. A Euclidean distance is the total distance between two points in a three-dimensional space. While breaking this distance down into (the vectors of) its three axes can offer additional insight into its clinical relevance, it is important to note that the Euclidean distance is larger than the values (vector length) of its three individual axes. For instance, considering the largest Euclidean distance observed in the TLNA group (4.55 mm), the deviation in any single direction, such as towards the pons, which lies axially and caudally to the trigeminal cistern, is less than this total distance. This implies that when using the TLNA and targeting the center of the trigeminal plexus at a safe distance (approximately 5 mm) from its dural boundaries, the likelihood of inadvertently puncturing an incorrect anatomical structure is negligible. Although we are confident that the described technical setup enables consistent targeting of the equine trigeminal cistern, we would still recommend verifying the needle tip’s precise location using an intraoperative CBCT scan before administering glycerol in a clinical scenario.

Numerous important neural and vascular structures surround the equine larynx and guttural pouches. Iatrogenic damage to these structures can be of major clinical relevance. Furthermore, perforation of the respiratory epithelial lining of the guttural pouch mucosa invariably results in contamination of the needle. In the worst-case scenario, such contamination could introduce pathogens into the intracranial space when puncturing the trigeminal cistern. Indeed, suppurative meningitis has been reported as a complication following glycerol rhizotomy in horses (13). Although the TLNA bypasses the equine larynx and the guttural pouch, focal perforation of the roof of the guttural pouch was observed in one specimen in the present cadaver study. Based on the retrospective analysis, we conclude that the planned trajectory had been positioned too close to the roof of the guttural pouch, highlighting the importance of cautious preoperative planning and critical evaluation of the trajectory using the probe’s eye function of the navigation system. A trajectory that is planned too ventrally not only increases the risk of perforating the roof of the guttural pouch but may also result in laceration of the maxillary artery or maxillary vein. A too dorsally positioned trajectory, on the other hand, could cause the needle to collide with the axial aspect of the mandibular condylar process, thereby impeding needle advancement or resulting in needle bending, ultimately compromising surgical accuracy. Thus, the lateral pterygoid muscle serves as a useful but narrow corridor to approach the ventrolateral aspect of the cranium, bordered by critical neurovascular structures. Intraoperative visualization of the roof of the guttural pouch using a flexible endoscope could enable early recognition of an inappropriate trajectory of the navigated needle, thus potentially further reducing the risk of laceration of the maxillary or internal carotid arteries.

In humans, perforation of the ICA with the injecting needle can result in serious complications, including pseudoaneurysm formation, caroticocavernous fistula, ICA rupture, and potentially life-threatening arterial hemorrhage (28). Unlike in horses, the ICA of humans does not travel through the foramen lacerum but instead passes through the carotid canal within the temporal bone to enter the cranial cavity. It then courses superior to the foramen lacerum, a segment referred to as the lacerum segment (29). Inadvertent puncture of the ICA during percutaneous cannulation of the foramen ovale can occur at several anatomical locations: its extracranial portion at its entrance in the carotid canal, at the exocranial surface of the skull base in the foramen lacerum or at its intracranial portion, such as within the cavernous sinus (16). The minimal distances from the foramen ovale to the trigeminal ganglion and the intracranial portion of the ICA have been measured, and guidelines for safe advancement of the needle have been established (15, 16). In contrast, in horses, the ICA travels from the roof of the medial compartment of the guttural pouch through the foramen lacerum, perforating its cartilage plate through a slit-shaped opening located beneath its rostrolateral edge, and subsequently forms the sigmoid flexure - a segment located just ventral to the trigeminal cave (30). The only possible ventral and/or lateral access route to the trigeminal cistern is through the foramen lacerum. This inherently carries a high risk of lacerating the sigmoid flexure, which is further amplified with the ventral approach, where the needle must cross the center of the sigmoid flexure. Thus, even an accuracy aberration of only a few millimeters over a total trajectory length of approximately 20 cm would result in its perforation. In seven out of eight cadaveric specimens that underwent the VNA, hemorrhage was observed at the needle insertion site in the roof of the guttural pouch - most likely originating from the carotid artery or the sinus petrosus (13). The results of the present study suggest that this complication could be avoided by electing the TLNA, as this would result in a trajectory dorsally “flying over” the ICA and its sigmoid flexure to reach the trigeminal cistern (Figure 3).

The clinical relevance of the distribution of toluidine blue staining along the mandibular nerves - and by extrapolation of the glycerol during chemical rhizotomy - can only be speculated. This distribution pattern was unlikely caused by gravity, as the specimens were in a supine position during the injection. A recent microanatomical study observed a similar distribution of a polymer-based contrast agent along the peripheral branches of the trigeminal nerve on micro-CT after trigeminal cisternography. This distribution was predominantly noticed along the mandibular nerves, but also along the ophthalmic and maxillary nerves in some specimens (12). The ophthalmic and maxillary nerves have a purely sensory function, and prolonged contact with glycerol could lead to greater pain relief and improve outcome following rhizotomy. However, as with every manipulation of the trigeminal sensory pathway, this could also lead to hypesthesia or anesthesia dolorosa. In contrast, the mandibular nerve has both sensory and motor functions: it innervates the masticatory muscles (i.e., the masseter, medial and lateral pterygoid, and temporal muscles) as well as the tensor veli palatini muscle, which maintains tension in the rostral aspect of the soft palate and thereby expands the nasopharynx during inspiration. Although glycerol rhizotomy in humans can cause masseter muscle weakness, the incidence of this complication is relatively low, and it occurs less frequently than with balloon compression or radiofrequency rhizotomy (31, 32). This muscle weakness is typically transient, resolves spontaneously, and rarely causes clinically significant deficits. To date, the number of TMHS cases treated with glycerol rhizotomy reported in the literature is too low to draw definite conclusions about the clinical relevance of this potential complication in horses.

This study has several limitations, including a heterogeneous study population and a relatively small sample size. However, all the included horses were adult and of comparable size and weight. In addition, using neuronavigation for surgical planning and real-time intraoperative guidance allowed each surgical approach to be tailored to the individual anatomy rather than relying on fixed anatomical landmarks that may vary between breeds. Despite the small sample size, the advantages of the TLNA over the VNA in terms of safety, accuracy, and precision were clearly evident in the analyzed specimens.

Another limitation is the lack of postprocedural assessment of the integrity of the buccal branches of the facial nerve. In clinical cases, every effort should be made to avoid inadvertent injury to facial nerve branches and subsequent facial muscle dysfunction. This includes careful dissection and gentle manipulation of the tissue surrounding the buccal branches of the facial nerve.

The primary objective of the present study was to develop a safe and accurate approach to puncture the trigeminal cistern in horses. The high-resolution soft tissue visualization provided by MRI is a key component in achieving this goal, especially given the length of the approach in the equine patient and the high density of vital neurovascular structures in the chosen corridor. However, in contrast to the widely recognized value of CT imaging of the head in the diagnosis of TMHS (33), the diagnostic value of MRI in this disease remains uncertain. This uncertainty is mainly related to the insidious etiology of TMHS (2) and the infrequent observation of structural changes reported on MRI in affected horses (34). Nonetheless, the current veterinary literature provides only limited information on MRI findings in horses with TMHS. As MRI becomes more accessible in equine referral centers, it may gain importance, particularly for excluding structural (central) nervous pathologies that might contribute to TMHS. If MRI is included in the diagnostic workup of TMHS or used in planning a controlled rhizotomy, a 3D MP-RAGE sequence should always be acquired, as it provides critical information for neuronavigation. For optimal visualization of blood vessels in live animals, T1-weighted sequences augmented with intravascular injection of gadolinium and time-of-flight magnetic resonance angiography are preferably performed (35).

The TLNA can be performed in either lateral, dorsal, or sternal recumbency, while the VNA may not be feasible in sternal recumbency. The trigeminal porus opens dorsally and is oriented obliquely from craniodorsal to caudoventral. Sternal recumbency may prevent premature caudal migration of glycerol into the caudal fossa due to gravity, promoting its pooling in the trigeminal cistern and thereby prolonging the contact time with ganglionic tissue. In humans, various postinjection patient positioning practices have been reported to prolong glycerol contact time with ganglionic tissue (36). The most frequently recommended position is sitting upright with the head flexed, i.e., bent forward so that the chin moves toward the chest, for 1 to 2 h. The optimal head position in horses after injection to maximize contact time has yet to be determined.

In conclusion, the TLNA allowed accurate and precise minimally invasive puncture of the trigeminal cistern in cadaveric specimens of horses. The injection procedure presents significant technical challenges due to the length of the surgical trajectory and the narrow corridor that is lined by important neurovascular anatomy. The use of highly specialized instrumentation and advanced 3D imaging is essential to achieve satisfactory accuracy and precision in needle placement within the trigeminal cistern while avoiding iatrogenic injury to the major neurovascular structures of the head. Despite all technical efforts, it must be stressed that a rhizotomy procedure involves the selective ablation of a central neural structure, which inherently carries a non-negligible risk of morbidity. At present, and until further evidence supports its broader clinical application, this ablative approach should be reserved for very specific cases. These include horses exhibiting severe trigeminal-mediated headshaking even at rest and across different environments, those with a severely compromised well-being, those unresponsive to all available therapeutic and management options, and only after other potential causes of headshaking have been thoroughly excluded. In combination with the recently published information about the microanatomy of the equine trigeminal cistern (12), the results of the present study may lay the foundation for the development of a controlled rhizotomy in horses suffering from severe TMHS refractory to medical treatment- an unresolved and serious animal welfare issue.

## Data Availability

The raw data supporting the conclusions of this article will be made available by the authors, without undue reservation.
